# On the journey to competency-based assessment for safe patient care

**DOI:** 10.3205/zma000989

**Published:** 2015-10-15

**Authors:** Jana Jünger, Ara Tekian, John J. Norcini

**Affiliations:** 1 Universität Heidelberg, Medizinische Fakultät, Kompetenzzentrum Prüfungen in der Medizin Baden-Württemberg, Heidelberg, Deutschland; 2 Univerity of Illinois at Chicago, Chicago, USA; 3 Foundation for Advancement of International Medical Education and Research (FAIMER), Philadelphia, USA

## Editorial

Over the past few decades there has been an international movement towards competency-based medical education (CBME). Germany has participated in this development and recently created the NKLM, which is a catalogue of the learning objectives for medical education [http://www.nklm.de and [http://www.nklz.de] citied 2015-09-18], [1]. The recommendations of the German Council for Science and Humanities regarding the further development of medical studies in Germany state that modified teaching and learning content should also be reflected in assessment [2]. The aims of this movement are to ensure that the graduates of our medical faculties have the necessary knowledge, skills, and attitudes to provide excellent patient care. 

Implementation of CBME requires appropriate alignment of the learning objectives specified in the NKLM with the curriculum, particularly with the instructional methods and assessment strategies. A number of institutions have been experimenting with ways of making the appropriate changes within the resources available. 

These exciting developments have been driven by enlightened initiatives in association with the German community of medical educators and it has generated a new culture for the academy. Central to CBME is the ability to determine when students have achieved the competencies specified in the NKLM and that requires advances in the way we do assessment. 

It is in this context that we have assembled the papers for this special issue. One critical feature of this new culture is the increased involvement of the students. An example of this is the article by Wagener et al. that reports the results of student-driven competency-based progress test [3]. Not only did the students create the material across faculties for this assessment, but it also informs them about their progression during their studies in light of the NKLM-competencies. One aspect of the culture change is an increased emphasis on using assessment to support learning. For example the article by Profanter et al. focuses on workplace-based assessment which is intended to provide feedback tailored to the strengths and weaknesses of the students [4]. As a result, the teachers modified their educational activities to enhance student learning. 

Students are often concerned about the fairness in their assessment. One area of on-going concern is the great variability in the grades assigned by various examiners. The paper by Schickler et al. demonstrates the importance of rater training [5]. This calibration of the examiners is important in oral examinations, especially where the stakes are high. It is clear that examiner training is an essential aspect of quality assurance.

The paper by Tekian and Norcini focuses on setting standards (i.e., selecting the pass-fail score) for examinations [6]. This topic has been largely ignored in Germany and it is essential to fairness to students. Moreover, the assumption of a 60% standard has the effect of influencing the examination content by requiring the inclusion of material based on its ability to generate the appropriate pass rates rather than based on the importance of the content. Likewise, the paper by Möltner et al. addresses the accuracy and the consistency of the pass/fail decision for assessment comprised of multiple components [7]. The paper by Hochlehnert et al. demonstrates the importance and role of technology in improving the quality and efficiency of assessment [8]. 

As we go forward, it is important to look to our colleagues worldwide of how to improve assessment. The paper by Berendonk et al. describes the introduction of the clinical skills examination into the national licensing examination [9]. 

These are only a few of the topics in assessment that are addressed in this special issue. These studies provide the first steps in achieving the goal of implementing CBME based NKLM. As we go into the future, additional progress will require collaboration, networking, and sharing of resources. Assessment is the means for improving the quality of care provided by individual practitioners. The next step is to ensure the quality of the educational programs themselves.

## Competing interests

The authors declare that they have no competing interests.
